# Arid1a regulates neural stem/progenitor cell proliferation and differentiation during cortical development

**DOI:** 10.1111/cpr.13124

**Published:** 2021-09-25

**Authors:** Xiao Liu, Shang‐Kun Dai, Pei‐Pei Liu, Chang‐Mei Liu

**Affiliations:** ^1^ State Key Laboratory of Stem Cell and Reproductive Biology Institute of Zoology Chinese Academy of Sciences Beijing China; ^2^ Savaid Medical School University of Chinese Academy of Sciences Beijing China; ^3^ Institute for Stem Cell and Regeneration Chinese Academy of Sciences Beijing China; ^4^ Beijing Institute for Stem Cell and Regenerative Medicine Beijing China

**Keywords:** *Arid1a*, cerebral cortex, differentiation, neurogenesis, NSPCs, proliferation

## Abstract

**Objective:**

Neurodevelopmental diseases are common disorders caused by the disruption of essential neurodevelopmental processes. Recent human exome sequencing and genome‐wide association studies have shown that mutations in the subunits of the SWI/SNF (BAF) complex are risk factors for neurodevelopmental diseases. Clinical studies have found that ARID1A (BAF250a) is the most frequently mutated SWI/SNF gene and its mutations lead to mental retardation and microcephaly. However, the function of ARID1A in brain development and its underlying mechanisms still remain elusive.

**Methods:**

The present study used Cre/loxP system to generate an *Arid1a* conditional knockout mouse line. Cell proliferation, cell apoptosis and cell differentiation of NSPCs were studied by immunofluorescence staining. In addition, RNA‐seq and RT‐PCR were performed to dissect the molecular mechanisms of *Arid1a* underlying cortical neurogenesis. Finally, rescue experiments were conducted to evaluate the effects of *Neurod1* or *Fezf2* overexpression on the differentiation of NSPCs in vitro.

**Results:**

Conditional knockout of *Arid1a* reduces cortical thickness in the developing cortex. *Arid1a* loss of function inhibits the proliferation of radial glial cells, and increases cell death during late cortical development, and leads to dysregulated expression of genes associated with proliferation and differentiation. Overexpression of *Neurod1* or *Fezf2* in *Arid1a* cKO NSPCs rescues their neural differentiation defect in vitro.

**Conclusions:**

This study demonstrates for the first time that *Arid1a* plays an important role in regulating the proliferation and differentiation of NSPCs during cortical development, and proposes several gene candidates that are worth to understand the pathological mechanisms and to develop novel interventions of neurodevelopment disorders caused by *Arid1a* mutations.

## Graphical abstract

Although *Arid1a* mutations are closely related to mental retardation and microcephaly, the function of *Arid1a* in brain development and its underlying mechanisms still remain elusive. The present study demonstrates for the first time that *Arid1a* plays an important role in regulating the proliferation and differentiation of neural stem/progenitor cells during cortical development, and proposes several gene candidates that are worth to understand the pathological mechanisms and to develop novel interventions of neurodevelopment disorders caused by *Arid1a* mutations.

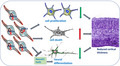

## 1 INTRODUCTION

The cerebral cortex development requires complex sequential processes that have to be precisely orchestrated.^1^ During the onset of cortical development, radial glial progenitor cells (RGCs), which derive from neuroepithelial cells, can divide symmetrically to expand the progenitor pool, whereas, in later stages, RGCs divide asymmetrically to directly generate neurons, but most of them indirectly give rise to neurons via intermediate progenitor cells (IPCs).^2^, ^3^ The generation of RGCs and IPCs results in the formation of two proliferative zones: the ventricular zone (VZ) and the adjacent subventricular zone (SVZ).^3^ During the development of cortex, cortical layering arises in an inside‐out manner as neural progenitors proliferate and differentiate into interneurons and projection neurons.^4^ Disruptions in the maintenance and/or the balance between proliferation and differentiation of neural progenitors are thought to result in many neurodevelopmental disorders.^5^, ^6^, ^7^

ATP‐dependent chromatin remodeling plays important roles during cortical neurogenesis.^8^ SWI/SNF complex, a class of ATP‐dependent chromatin remodelers, have been reported to interfere with the structure of chromatin, release of nucleosome‐bound DNA, mobilization of DNA along nucleosomes and displacement of histone dimers promoting nucleosome disassembly.^9^, ^10^, ^11^ In addition, recent genome‐wide studies indicate that it is involved in cellular processes such as cell proliferation and differentiation.^12^, ^13^ ARID1A (the AT‐rich interaction domain 1A, also known as BAF250a), the largest subunit of the SWI/SNF chromatin remodeling complex, has been reported that its mutations are closely related to Coffin–Siris syndrome (CSS), which is characterized by intellectual disability, growth deficiency and microcephaly.^14^, ^15^, ^16^ In early mouse embryos, ablation of *Arid1a* results in developmental arrest by severely inhibiting self‐renewal and promoting differentiation into primitive endoderm‐like cells.^17^ In the central nervous system (CNS), loss of *Arid1a* in neural crest cells (NCCs) leads to craniofacial defects in adult mice, including shortened snouts and low set ears, and these defects are more pronounced following homozygous, which is similar to CSS.^18^ However, the biological functions and mechanisms of *Arid1a* in microcephaly and intellectual disability are still unknown.

Here, we generated *Arid1a* conditional knockout mice and found that deletion of *Arid1a* in forebrain neural stem/progenitor cells (NSPCs) results in thinner cortex. Loss of *Arid1a* decreases the number of deep‐layer cortical neurons and increases cell death during late embryonic cortical development. In addition, *Arid1a* deletion leads to a decrease in the proliferation of RGCs and an increase in the proliferation of IPCs in the developing cortex. Global transcriptome analysis after *Arid1a* deletion reveals dysregulated expression of genes that are associated with the proliferation and differentiation of NSPCs. Overexpression of *Neurod1* or *Fezf2* in *Arid1a* cKO NSPCs rescues the neural differentiation defect in vitro. These results together highlight the essential roles of *Arid1a* in cortical development and support that loss of function of *Arid1a* contributes to microcephaly.

## 2 MATERIALS AND METHODS

### 2.1 Mice

All mice used in the current study have a C57BL6 background. The *Arid1a* ^f/f^ mouse was a kind gift from Dr. Zhong Wang at University of Michigan and Dr. Chun‐sheng Han at the Institute of Zoology, Chinese Academy of Sciences. Mice were genotyped by PCR using primers (Forward, 5′‐TGGGCAGGAAAGAGTAATGG‐3′; Reverse, 5′‐AACACCACTTTCCCATAGGC‐3′) and conditions used by The Jackson Laboratory. The *Arid1a* ^f/f^ mice and Emx1‐cre mice (JAX Stock No. 005628) were crossed for the generation of *Arid1a* conditional knockout (cKO) mice. All experiments involving mice were approved by the Animal Committee of the Institute of Zoology, Chinese Academy of Sciences.

### 2.2 Tissues

We accurately obtain mouse embryos in the following ways: after 5 pm on the first day, we put *Arid1a* ^f/‐^: Emx1‐cre male mice and *Arid1a* ^f/f^ female mice together. The vagina of the female mouse was examined at 9 am the next day. When the vaginal suppository appeared, it was E0.5. Embryonic brains of E12.5, E14.5 and E16.5 were fixed in 4% paraformaldehyde overnight and dehydrated with 30% sucrose.

### 2.3 BrdU incorporation analysis

Pregnant mice were given intraperitoneal injections with 100 mg/kg BrdU based on the weight of the mouse; the concentration of stock BrdU is 10 mg/ml (Sigma; B5002‐5G). Embryonic brains were harvested 2 h after BrdU pulsing at E12.5, E14.5 or E16.5.

### 2.4 Immunofluorescence staining

Brains were cut into 10‐µm‐thick cryosections. For immunofluorescence analyses, brain slices were washed three times in PBS (10 min each time). To detect BrdU incorporation, fixed brain slices were pre‐treated with 1 M HCl for 30 min at 37°C. The slices were then washed with 0.1 M borate buffer (pH 8.5) for 30 min (15 min each time) and with PBS for 30 min (10 min each time). Slices were fixed in 4% paraformaldehyde for 15 min at room temperature and washed three times in PBS (10 min each time). Next, the slices were blocked in a blocking solution (2% bovine serum albumin, 0.3% Triton X‐100 and 0.1% sodium azide) at room temperature for 2 h. The slices were then incubated with the primary antibodies (anti‐ARID1A, Sigma, HPA005456; anti‐Nestin, Aves Labs, NES; anti‐Tuj1, Biolegend, 801202; anti‐PAX6, Biolegend, 901301; anti‐BrdU, abcam, ab6326‐125; anti‐PHH3, Millipore, 09‐797; anti‐Ki67, Labvision, RM‐9106‐S1) diluted in the blocking solution at 4°C overnight and labelled using the appropriate secondary antibodies (Goat anti‐mouse 488, A11001; Goat anti‐rabbit 488, A11034; Goat anti‐rabbit 568, A11011; Goat anti‐rat 568, A11077; Goat anti‐chicken 488, A11039; Donkey anti‐goat 568, A11057) at room temperature for 2 h.

### 2.5 Western blot analysis

The total protein of cortical tissues was extracted using RIPA buffer, and the protein concentration was defined using BCA protein assay kit (Biomed, P0012). Western blotting was conducted according to published approaches.^19^ Briefly, the membranes were blocked in 3% milk in TBS‐T (Tris‐buffered saline with 0.1% Tween 20) and incubated with the primary antibodies (anti‐β‐actin, Sigma, A5441, 1:5000; anti‐ARID1A, Sigma, HPA005456, 1:1000; anti‐Flag, CST, 14793, 1:1000) at 4°C overnight. The membranes were then washed in TBS‐T for 10 min, three times and incubated with the secondary antibodies (anti‐rabbit HRP, C1309, 1:3000; anti‐mouse HRP, C2225, 1:3000) at room temperature for 2 h. Signal detection was conducted by the ECL system (Pierce) and Tanon‐5200 Chemiluminescent Imaging System (Tanon, China, Shanghai). The relative protein levels among the samples using the β‐actin density as an internal loading control were compared.

### 2.6 RNA‐seq and RT‐PCR

The total RNA was extracted from E16.5 *Arid1a* WT or cKO forebrains according to procedures with TRIzol reagent (Invitrogen, 15596018). After quality quantification, the total RNA was converted to cDNA library and analysed by Illumina HiSeq 2500 platform. The RNA‐seq data are available in SRA with accession number PRJNA726035.

For RT‐PCR analysis, total RNA was transcribed into cDNA using TransScript One‐Step gDNA Removal and cDNA synthesis Kit (TransGen Biotech, Beijing, China). Then, cDNA was quantified by using Hieff® qPCR SYBR® Green Master Mix in a 20 μl reaction system according to instructions. The PCR steps were performed as follows: initial pre‐denaturation at 95°C for 5 min, amplification for 45 cycles of 94°C for 10 s, 60°C for 30 s at, 72°C for 30 s, and final extension at 72°C for 10 min. All samples were run in triplicate. The analysis of relative gene expression was performed by the 2‐ΔΔCT method. GAPDH was used as endogenous control to normalize the RNA content of samples. All the primers used for RT‐PCR in this study are listed in Table S1.

### 2.7 Construction of plasmids

To generate the Neurod1‐OE plasmid or Fezf2‐OE plasmid, a pair of primers were annealed, and the product was inserted into the *Nhe1*/*EcoR1* restriction sites of the CD511 vector. The following primers were used: Neurod1‐OE‐F, 5′‐GACGGCTAGC GCCACCATGACCAAATCATACAGCGAGAGCGGGC‐3′; Neurod1‐OE‐R, 5′‐CCGGAATTCCTTGTCATCGTCGTCCTTGTAATCCTAATCGTGAAAGATGGCATTAAGCTGGGC‐3′; Fezf2‐OE‐F, 5′‐GACGGCTAGCGCCACC ATGGCCAGCTCAGCTTCCCTGGAGACCA‐3′; Fezf2‐OE‐R, 5′‐CCGGAATTCCTTGTCATCGTCGTCCTTGTAATCTCAGCTCTGAACTGTCCTGGCTAGGTCC‐3′.

### 2.8 Lentivirus production

Lentivirus production was performed as described previously.^19^ Lentiviral vector and packaging plasmid were co‐transfected into 293T cells through polyethylenimine.^20^ After transduction, the serum‐free medium was replaced by fresh culture medium after 6 h. The medium was collected at 48, 72 and 96 h post‐transduction. Lentivirus were then concentrated with an ultracentrifuge at 38,000 g for 2 h at 4°C and dissolved in 1 × PBS.

### 2.9 Differentiation analyses of cultured NSPCs

NSPCs were isolated from Arid1a WT and cKO forebrain at E16.5 as described previously.^19^, ^21^ Briefly, brain tissues were digested with TrypLE Express (Gibco, #12604013) in a 5% CO2 incubator at 37°C for 8 min. Then, 1 ml of DMEM/F‐12 containing 10% FBS, 1% GlutaMAX (Gibco, 35050061) and 1% Antibiotic‐Antimycotic (GIBCO, #15240‐062) was added into each sample to stop digestion. Single cells were obtained though scattering by repetitive pipetting and passing through 70 µm cell strainer. The single‐cell suspension was cultured with DMEM/F‐12 medium containing 1% N2 supplement (GIBCO, #17502‐048), 1% Antibiotic‐Antimycotic, 20 ng/ml basic fibroblast growth factor (FGF‐2, PeproTech), 20 ng/ml epidermal growth factor (EGF, PeproTech) in a 5% CO2 incubator at 37°C.

Differentiation of NSPCs assays was performed as previously.^19^, ^21^, ^22^ NSPCs were seeded on poly‐L‐ornithine/laminin‐coated coverslips at a density of 2 × 10^5^ cells/well. Following lentiviral infection for 48 h, NSPCs were incubated with DMEM/F‐12 medium containing 1% N2 supplement (GIBCO, #17502‐048), 1% Antibiotic‐Antimycotic, 5 µM forskolin (FSK, Sigma‐Aldrich, #F‐6886) and 1 µM retinoic acid (RA, Sigma‐Aldrich, #R‐2625) for 3 days. NSPCs were then fixed with 4% paraformaldehyde for 20 min and stained with Tuj1 antibody.

### 2.10 Microscope imaging

Confocal images were acquired using Zeiss LSM 710 and LSM880 Fast Airyscan confocal microscopes and analysed by ZEN software.

### 2.11 Statistical analysis

Experiments were conducted in at least three biological replicates for each group. Immunostaining quantification analysis was performed with ImageJ. Positive cells were counted by a rectangle with a width of 100 µm on the cortex, and at least 10 sections were examined for each embryo. For statistical analyses, unpaired two‐tailed Student's *t* tests were performed using GraphPad Prism software. Statistical significance was defined as *p < 0.05, **p < 0.01 and ***p < 0.001. Unless otherwise indicated, all data are presented as mean ± SEM.

## 3 RESULTS

### 3.1 Loss of *Arid1a* reduces cortical thickness in the developing cortex

**FIGURE 1:**
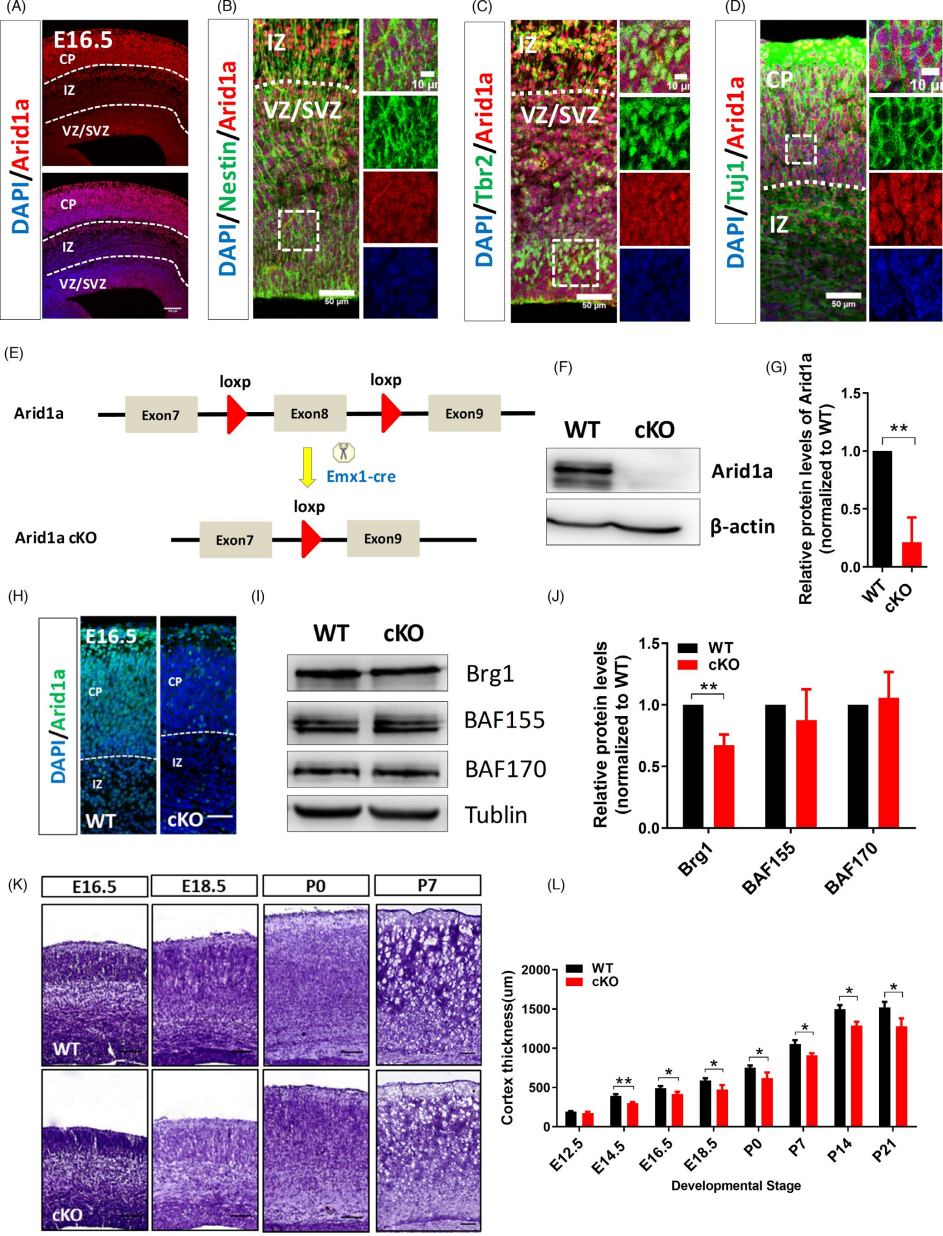
Loss of *Arid1a* reduces cortical thickness in the developing cortex. (A) Immunofluorescence staining for *Arid1a* in the E16.5 cortex. Embryonic brain sections were immunostained with anti‐Arid1a antibody. Scale bar, 100 µm. (B–D) *Arid1a* is expressed in Nestin^+^ NSPCs, Tbr2^+^ intermediate progenitors and Tuj1^+^ neurons in the E16.5 cortex. (E) Schematic diagram for the generation of *Arid1a* conditional knockout mice (cKO). The mouse line with loxP sites inserted on both sides of exon 8 of *Arid1a* gene was crossed with the mouse line to create *Arid1a* cKO mice. (F, G) Western blot and quantification results showed that the *Arid1a* protein level was significantly reduced in the cortex of *Arid1a* cKO mice. (H) Immunofluorescence staining confirmed that *Arid1a* is almost undetectable in the cortex of *Arid1a* cKO mice. (I, J) Western blot and quantification demonstrated that the Brg1 protein level in the cortex of *Arid1a* cKO mice was significantly reduced, and BAF155 and BAF170 were almost no change. (K) The reduced cortical thickness of *Arid1a* cKO mice was observed at E16.5, E18.5, P0 and P7 by Nissl staining. (L) Quantification of cortical thickness at different developmental stages. WT, *n* = 3; cKO, *n* = 3. Scale bar, 50 µm. *p < 0.05, **p < 0.01, ***p < 0.001.

To investigate the function of *Arid1a* in the development of CNS, we first tested the expression pattern of *Arid1a* in the brain of E16.5 embryos. The immunostaining results showed that *Arid1a* was ubiquitously expressed in the nucleus of NSPCs in the VZ/SVZ and of neurons in the cortical plate (CP) (Figure 1A). Co‐immunostaining of ARID1A with the NSPCs marker Nestin confirmed the expression of *Arid1a* in NSPCs (Figure 1B). In addition, *Arid1a* was also expressed in Tbr2^+^ intermediate progenitors (Figure 1C). Furthermore, *Arid1a* was also highly expressed in Tuj1^+^ neurons (Figure 1D). Taken together, these results clearly demonstrated that *Arid1a* is widely expressed in the cortex during forebrain development, suggesting that *Arid1a* may play a pivotal role in regulating the development of embryonic cerebral cortex.

To examine the roles of *Arid1a* in cortical development, we generated *Arid1a* cKO mice by crossing *Arid1a* ^f/f^ mice with Emx1‐Cre mice that drive cortex‐specific Cre expression beginning at E9.5 (Figure 1E). The results from immunofluorescence staining and Western blot showed that *Arid1a* was successfully deleted in the forebrain as its protein level was significantly reduced in *Arid1a* cKO mice (Figure 1F‐H). Moreover, the expression of Brg1, the central ATPase subunit of SWI/SNF, was significantly down‐regulated after *Arid1a* deletion (Figure 1I,J). However, the expression of BAF155 and BAF170, another two core subunits of SWI/SNF, was no difference between WT and cKO mice (Figure 1I,J). Interestingly, *Arid1a* cKO mice had a reduced cortical thickness from E14.5 to P21 compared to WT mice (Figure 1K,L), indicating that *Arid1a* loss of function did affect cortical development.

### 3.2 Deletion of *Arid1a* results in abnormal differentiation of deep‐layer cortical neurons

**FIGURE 2:**
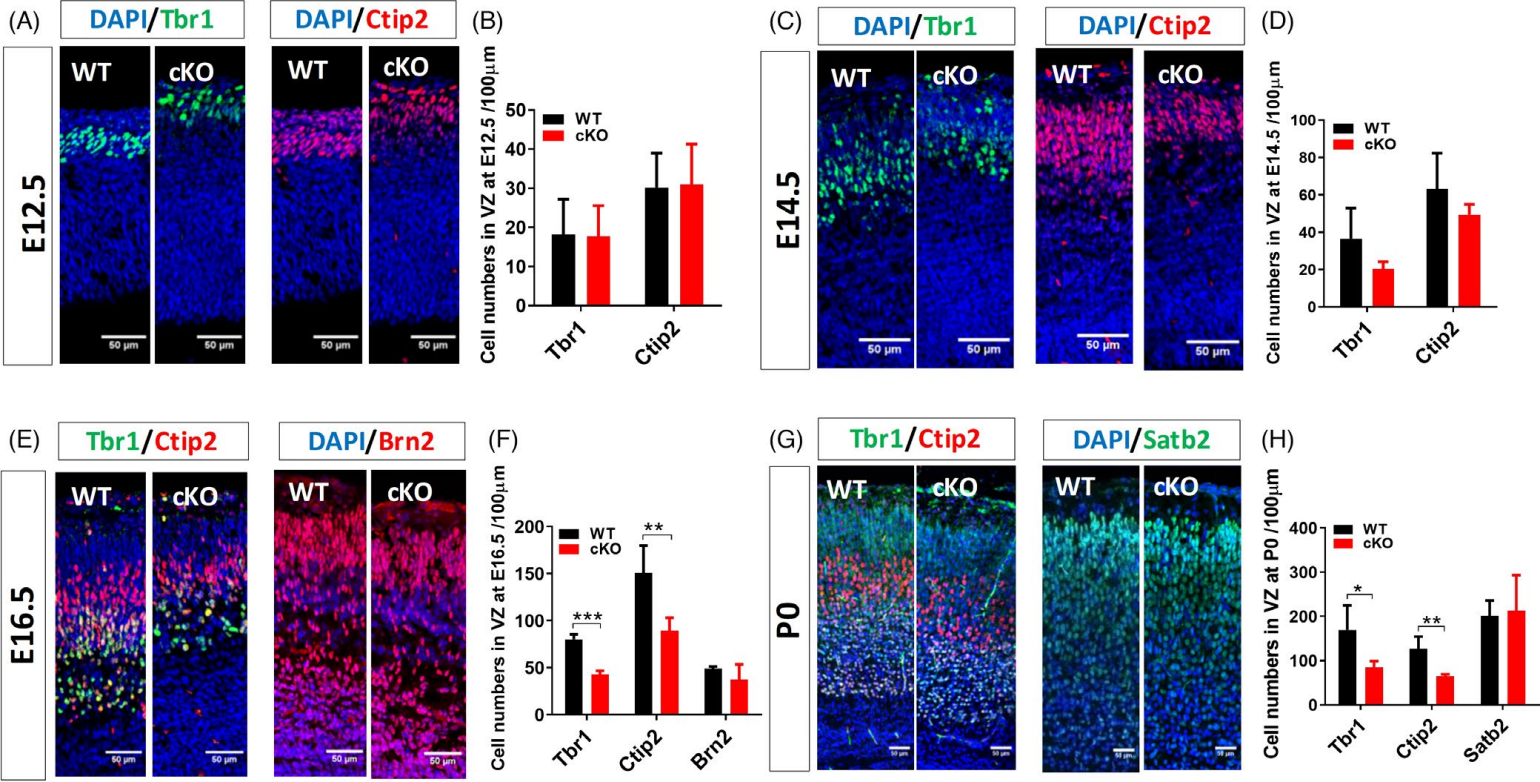
A decrease in the number of deep‐layer cortical neurons in the developing cortex of *Arid1a* cKO mice. (A, B) Immunofluorescence staining showed that the absence of *Arid1a* in NSPCs did not affect the formation of cortical layers V and VI at E12.5. (C, D) At E14.5, the knockout of *Arid1a* led to a trend of decline in the number of deep‐layer neurons. (E, F) At E16.5, the knockout of *Arid1a* resulted in a significant decrease in the number of deep‐layer neurons, while the number of neurons in the superficial layer did not change significantly. (G, H) The number of deep‐layer neurons is also reduced in *Arid1a* cKO mice after birth, and the number of superficial layer neurons remains unchanged. WT, *n* = 3; cKO, *n* = 3. Scale bar, 50μm. *p < 0.05, **p < 0.01, ***p < 0.001.

A previous report has shown that loss of *Arid1a* in hematopoietic stem cells impairs the differentiation of both myeloid and lymphoid lineages in hematopoiesis.^23^ To test whether the reduced cortical thickness in *Arid1a* cKO mice was caused by the deficit in neural differentiation of cortical NSPCs, we firstly used the markers Tbr1 and Ctip2 to label layer VI and layer V neurons in the cortex, respectively. At E12.5, the *Arid1a* cKO and WT cortices had equal numbers of layer V and layer VI neurons (Figure 2A,B). At E14.5, approximately the midpoint of cortical neurogenesis, the numbers of layer V and layer VI neurons started to decrease in *Arid1a* cKO mice compared to that in WT littermates (Figure 2C,D). At E16.5 there was a significant reduction in the number of layers V/VI neurons in the *Arid1a* cKO cortex, while there was no difference in the numbers of upper‐layer (layers II–IV) neurons as well as Brn2‐expressing later‐born neurons between *Arid1a* cKO and WT mice (Figure 2E,F). After birth, the number of layers V/VI neurons was still decreased, but the number of layers II–IV neurons remained unaltered in *Arid1a* cKO cortex (Figure 2G,H). Therefore, deletion of *Arid1a* results in reduced production of deep‐layer cortical neurons during the late embryonic development, which might contribute to the reduced cortical thickness in *Arid1a* cKO mice.

Next, we examined cell death in the cortex of *Arid1a* WT and cKO mice by TUNEL assay. We observed a 5‐fold increase in TUNEL^+^ cells at E12.5 and E14.5 and a 4‐fold increase in TUNEL^+^ cells at E16.5 upon *Arid1a* knockout (Figure S1A,B). These results suggested that *Arid1a* deficiency increased cell death during cortical development, which might be another possible cause for thinner cortex in *Arid1a* cKO forebrain.

### 3.3 *Arid1a* regulates the numbers of RGCs and IPCs in the developing cortex

**FIGURE 3:**
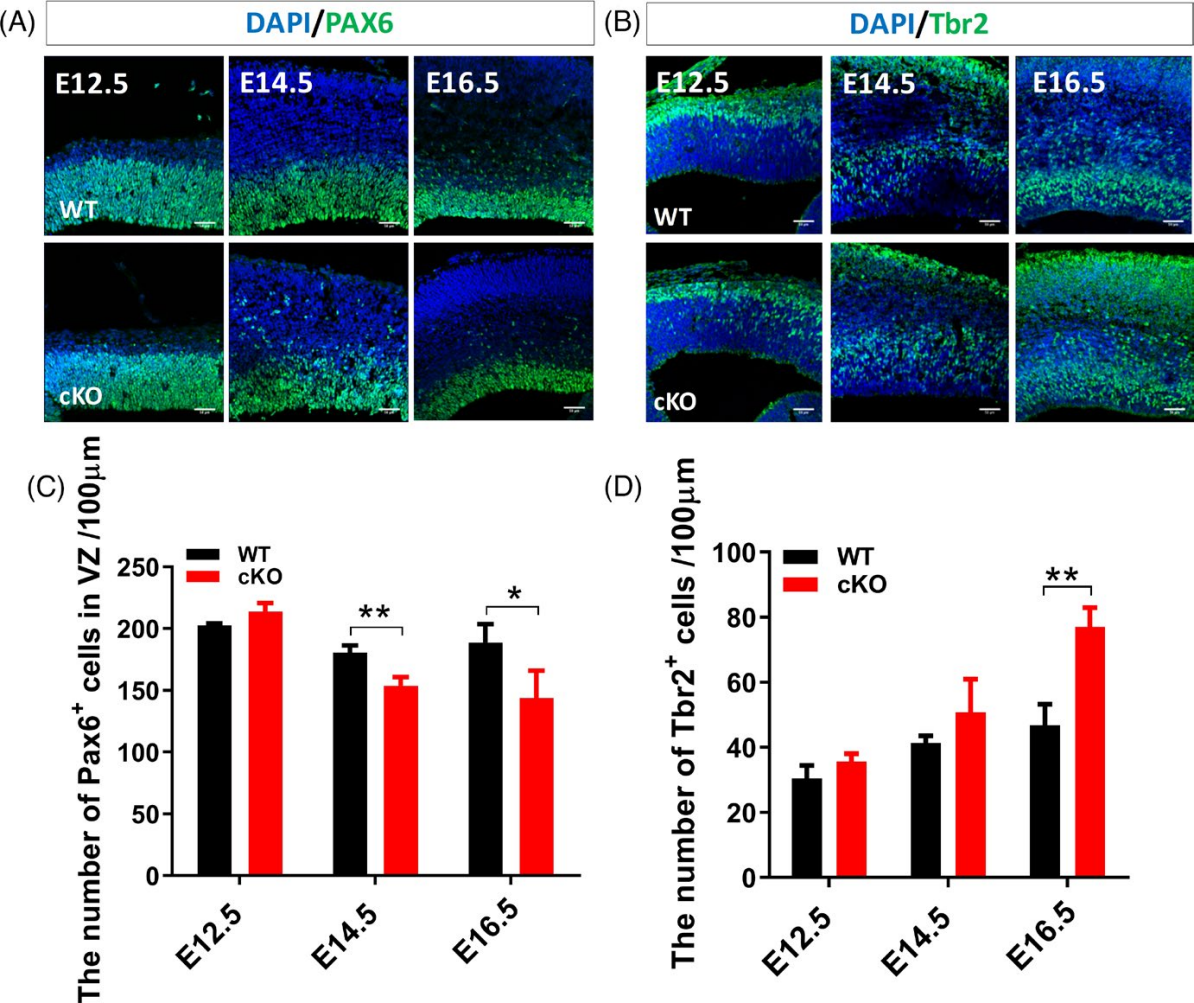
*Arid1a* regulates the numbers of RGCs and IPCs in the developing cortex. (A) Immunofluorescence staining of PAX6 at E12.5, E14.5 and E16.5. (B) The number of PAX6^+^ cells is not changed significantly in *Arid1a* cKO mice at E12.5, but is significantly reduced at E14.5 and E16.5. (C) Immunofluorescence staining of Tbr2 at E12.5, E14.5 and E16.5. (D) There is no significant change in the number of Tbr2^+^ cells at either E12.5 or E14.5; however, the number of Tbr2^+^ cells is increased significantly in *Arid1a* cKO mice at E16.5. WT: *n* = 3; cKO: *n* = 3. Scale bar, 50μm. *p < 0.05, **p < 0.01.

RGCs and IPCs are two classes of progenitors during cortical neurogenesis. In general, most RGCs divide asymmetrically to give rise to neurons via IPCs.^2^ To investigate the role of *Arid1a* in RGCs and IPCs, we performed immunostaining of PAX6 and Tbr2 to label RGCs and IPCs, respectively. At E12.5, the number of PAX6^+^ cells in the cortex was no difference between *Arid1a* cKO and WT littermates. At E14.5 and E16.5, however, there was a significant decrease in the number of PAX6^+^ cells in *Arid1a* cKO cortex (Figure 3A,B). In contrast, the number of TBR2^+^ cells, which are generated from PAX6^+^ cells, was significantly increased in *Arid1a* cKO cortex at E16.5 (Figure 3C,D). These results indicated that *Arid1a* may regulate the transformation of RGCs into IPCs and the size of the progenitor cell pool.

### 3.4 *Arid1a* promotes the proliferation of RGCs

**FIGURE 4:**
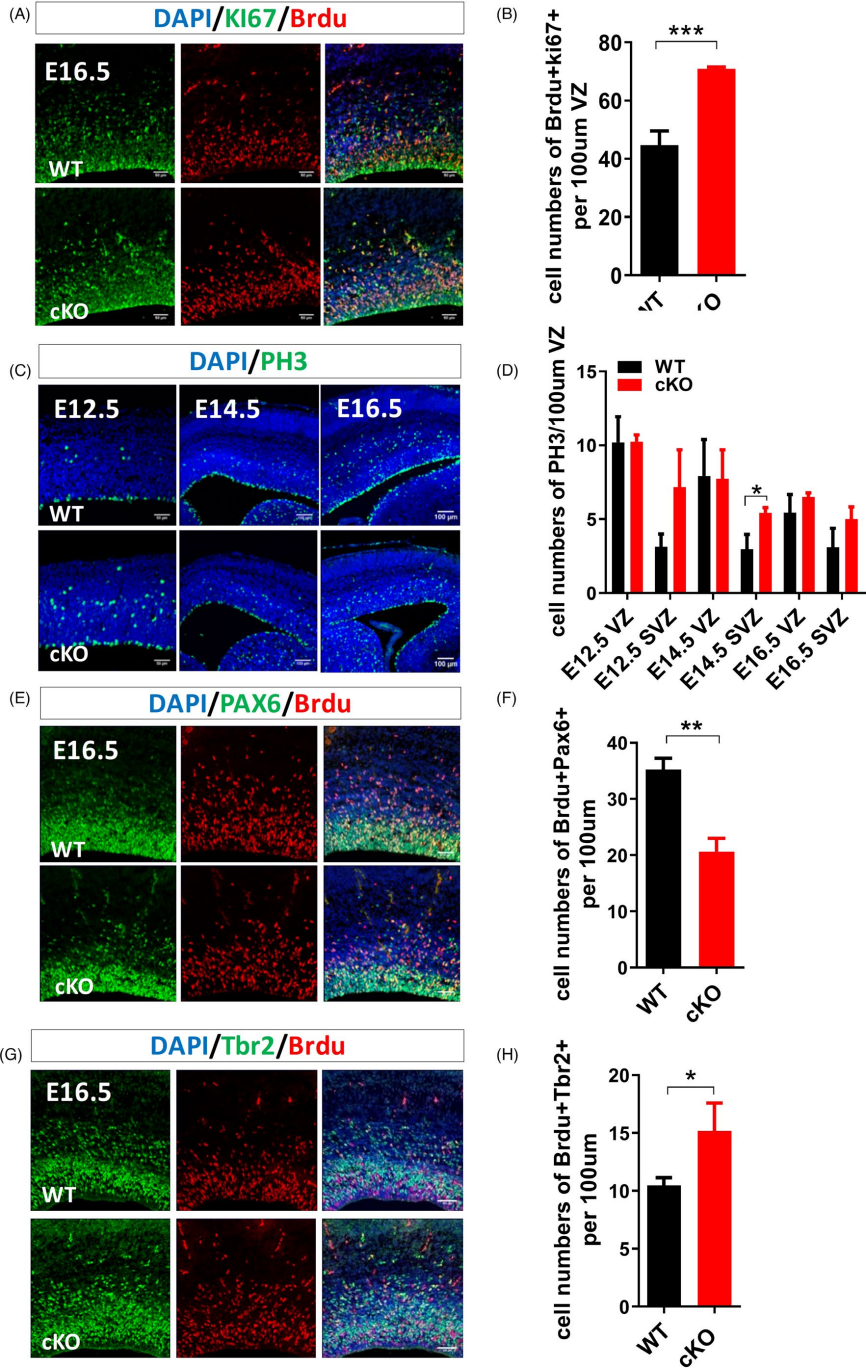
*Arid1a* cKO inhibits the proliferation of RGCs in the developing cortex. (A) Representative images of BrdU (red) and Ki67 (green) immunofluorescence staining of *Arid1a* WT and cKO brain sections at E16.5. (B) Quantitative analysis of BrdU^+^Ki67^+^ cell numbers in the cerebral cortex of *Arid1a* WT and cKO mice at E16.5. (C) Representative images of PH3 (green) immunofluorescence staining on brain sections of *Arid1a* WT and cKO mice at E12.5, E14.5 and E16.5. (D) Quantitative analysis of the numbers of PH3‐positive cells in the cerebral cortex of *Arid1a* WT and cKO mice at different developmental stages. (E) Representative images of BrdU (red) and PAX6 (green) immunofluorescence staining of *Arid1a* WT and cKO brain sections at E16.5. (F) Quantitative analysis of BrdU^+^PAX6^+^ cell numbers in the cerebral cortex of *Arid1a* WT and cKO mice at E16.5. (G) Representative images of BrdU (red) and Tbr2 (green) immunofluorescence staining of *Arid1a* WT and cKO brain sections at E16.5. (H) Quantitative analysis of BrdU^+^Tbr2^+^ cell numbers in the cerebral cortex of *Arid1a* WT and cKO mice at E16.5. WT: *n* = 3; cKO: *n* = 3. Scale bar, 50μm. *p < 0.05, **p < 0.01, ***p < 0.001.

To examine the role of *Arid1a* in the proliferation of NSPCs, E12.5, E14.5 or E16.5 pregnant mice were intraperitoneal injected with bromodeoxyuridine (BrdU) to label S phase dividing cells, and animals were euthanized 2 h later. *Arid1a* cKO resulted in a significant increase in BrdU incorporation at E16.5 (Figure 4A,B; Figure S2A‐C). Next, we used the phosphorylated histone 3 (PH3) to specifically label mitotic M‐phase cells, and detected PH3‐positive mitotic cells in both VZ and SVZ of the cortex (Figure 4C,D). Mitotic cells at the VZ surface are characteristic of radial glial cells, while those mitotic cells at SVZ are characterized as IPCs.^24^, ^25^ The number of PH3‐positive cells in the VZ had no significant difference between *Arid1a* cKO and WT mice during cortical development; however, the number of PH3‐positive cells in the SVZ was increased significantly in *Arid1a* cKO mice at E14.5 (Figure 4C,D).

To further reveal the role of Arid1a in RGCs and IPCs, the proliferation of RGCs and IPCs was assessed using BrdU labelling 2 h before the pregnant mice were euthanized at E16.5. We found that the proliferation of RGCs (PAX6^+^BrdU^+^) cells was reduced significantly, whereas the proliferation of IPCs (Tbr2^+^BrdU^+^) was significantly increased in *Arid1a* cKO mice compared to that in WT mice (Figure 4E‐H). These data support the idea that *Arid1a* promotes the proliferation of RGCs but decreases the proliferation of IPCs in the VZ/SVZ at E16.5.

### 3.5 *Arid1a* deletion leads to dysregulated expression of genes related to proliferation and differentiation of NSPCs

**FIGURE 5:**
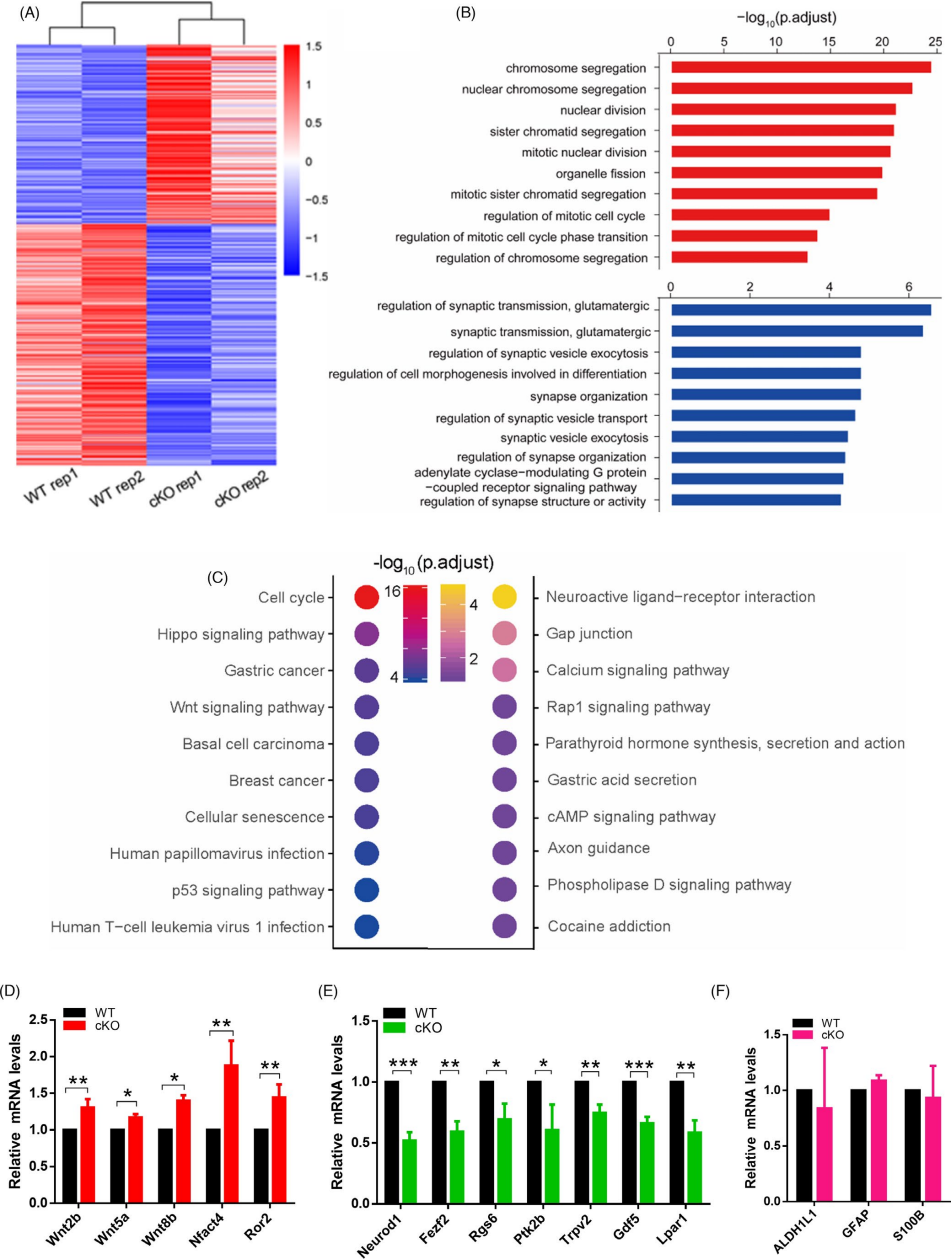
*Arid1a* deletion leads to dysregulated expression of genes associated with proliferation and differentiation. (A) Heatmap showing dysregulated genes in E16.5 *Arid1a* cKO cortical tissues, of which 239 genes are up‐regulated and 315 genes are down‐regulated (*p*‐value < 0.05; | log2foldchange |> 0.5). *n* = 2. (B) Top significantly enriched GO terms of dysregulated genes in the E16.5 cortex following *Arid1a* deletion. (C) KEGG pathway analysis found that up‐regulated genes in *Arid1a* cKO are enriched in Hippo signalling pathway and in Wnt signalling pathway. (D) qPCR validated the up‐regulation of genes related to the Wnt signalling pathway. (E) qPCR confirmed the down‐regulation of genes related to neural differentiation. (F) qPCR showed that the normal expression of glial cell‐enriched genes in *Arid1a* cKO cortex. *n* = 3; *p < 0.05, **p < 0.01, ***p < 0.001.

To further understand the molecular mechanism underlying *Arid1a* modulating cortical neurogenesis, we performed RNA sequencing to investigate the transcriptome differences in the cerebral cortex between *Arid1a* WT and cKO mice at E16.5. Genome‐wide analyses identified a large number of differentially expressed genes (DEGs) in *Arid1a* cKO mice compared with control littermates, of which 239 genes were up‐regulated and 319 genes were down‐regulated (*p*‐value < 0.05; | log2foldchange |> 0.5) (Figure 5A). Gene Ontology (GO) analysis showed that the up‐regulated genes were enriched in the functional terms of Chromosome Segregation, Nuclear Division and Regulation of Mitotic Cell Cycle, indicating that *Arid1a* is essential for the proliferation of NSPCs (Figure 5B). The down‐regulated genes were enriched in the functional terms related to Synaptic Transmission and Synapse Organization, indicating the potential regulation of *Arid1a* in the process of neuronal differentiation (Figure 5B). Furthermore, KEGG pathway analysis demonstrated that up‐regulated genes in *Arid1a* cKO were mainly enriched in Hippo signalling and Wnt signalling pathways (Figure 5C).

Next, we then focused on a subset of top DEGs on the list of GO terms and validated their expression changes by RT‐PCR (Figure 5D‐F). Consistently, RT‐PCR analyses confirmed that the expressions of Wnt signalling genes such as *Wnt2b*, *Wnt5a*, *Wnt8b*, *Nfact4 and Ror2* were up‐regulated in *Arid1a* cKO cortex (Figure 5D). In contrast, the expressions of *Fezf2*, *Rgs6*, *Ptk2b*, *Trpv2*, *Gdf5 and Lpar1*, which were involved in forebrain neuron differentiation, positive regulation of neuron differentiation, cerebral cortex neuron differentiation and forebrain generation of neurons, were significantly decreased in *Arid1a* cKO cortex (Figure 5E). Besides, RT‐PCR results showed that the expression of astrocytic genes (ALDH1L1, GFAP and S100β) did not alter after *Arid1a* cKO (Figure 5F). Taken together, *Arid1a* deletion leads to dysregulated expression of genes related to proliferation and differentiation of NSPCs.

### 3.6 Overexpression of *Neurod1* or *Fezf2* in *Arid1a* cKO NSPCs rescues the neural differentiation defect in vitro

To determine the downstream targets of *Arid1a*, we filtered *Fezf2* and *Neurod1* out for further exploration from the above expression‐validated genes associated with proliferation and differentiation of NSPCs. *Fezf2* and *Neurod1* are well‐known regulators of neuronal differentiation.^26^, ^27^, ^28^, ^29^ To explore whether *Arid1a* directly regulated NSPCs differentiation through *Neurod1* or *Fezf2*, we performed binding analysis with publicly available ChIP‐seq data for *Arid1a* from mouse embryonic stem cells^30^ and human embryonic stem cells.^31^ ChIP‐seq analysis indicates that there exist *Arid1a*‐binding peaks on the regions of *Neurod1* or *Fezf2* loci, suggesting that *Neurod1* or *Fezf2* might be the direct targets of *Arid1a* (Figure S3A,B). Further analysis of accessibility peaks in *Arid1a* WT and KO in mouse retinal ganglion cells (RGCs) with publicly available *Arid1a* ATAC‐seq data showed that *Arid1a* deletion in RGCs led to a dramatic decrease in the activity of neurogenic genes, including *Neurod1* and *Fezf2* (Figure S3C).^31^ However, publicly available ChIP‐seq data for BRG1 from mouse E16.5 cortical neurons demonstrate that there are no *Brg1*‐binding enrichments on the regions of Neurod1 or Fezf2 loci (Figure S4A,B). These data suggest that *Arid1a* could bind to *Neurod1* and *Fezf2* and directly regulate the expression of the two genes.

**FIGURE 6:**
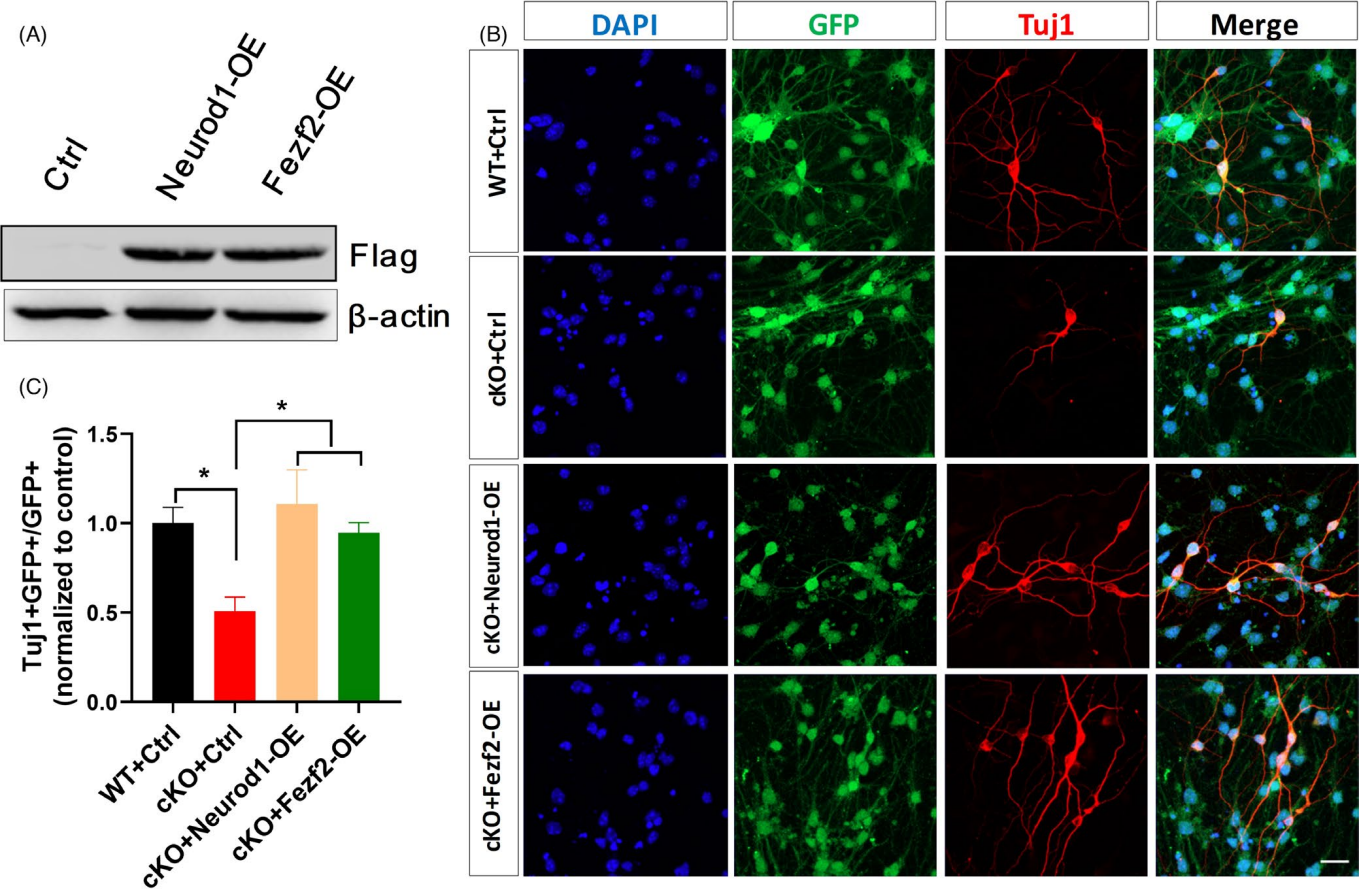
Overexpression of *Neurod1* or *Fezf2* in *Arid1a* cKO NSPCs rescues their neural differentiation defect in vitro. (A) Western blot for Flag in lysates of 293T cells transfected with the *Neurod1* overexpression vector, the *Fezf2* overexpression vector or an empty vector. (B) Representative images of Tuj1 immunostaining of E16.5 *Arid1a* cKO cortical NSPCs infected with Neurod1‐OE, Fezf2‐OE or control lentivirus. (C) Quantitative analysis demonstrated that overexpression of *Neurod1* or *Fezf2* enhanced the proportion of Tuj1+ cells differentiated from *Arid1a* cKO NSPCs. *n* = 3. Scale bar, 50 μm. *p < 0.05.

To further identify *Neurod1* and *Fezf2* are functional downstream targets of *Arid1a*, we firstly generated the lentiviral plasmids for overexpressing Neurod1(NeuroD1‐OE) and Fezf2 (Fezf2‐OE) respectively. The elevated protein levels of Neurod1 and Fezf2 were validated in 293T cells, which were transfected with lenti‐NeuroD1‐OE and lenti‐ Fezf2‐OE, respectively (Figure 6A). Secondly, we packaged lenti‐Neurod1‐OE virus and lenti‐Fezf2‐OE virus and infected cultured *Arid1a* cKO NSPCs to evaluate whether overexpression of *Neurod1* or *Fezf2* in Arid1a cKO NSPCs could rescue their neural differentiation defect. Indeed, our results showed that overexpression of *Neurod1* or *Fezf2* was sufficient to enhance the neural differentiation ability of *Arid1a* cKO NSPCs (Figure 6B,C). These results suggest that *Neurod1* and *Fezf2* are functional downstream targets of *Arid1a* during cortical development.

## 4 DISCUSSION

Neurogenesis is under the precise temporal and spatial control by many transcription factors.^32^, ^33^ Abnormal neurogenesis often results in neurodevelopmental disorders. Our study provides the first evidence that *Arid1a* plays an essential role in embryonic cortical neurogenesis. Conditional knockout of *Arid1a* decreases the generation of deep‐layer neurons and increases cell death, which results in a thinner cortex during late embryonic development. *Arid1a* loss of function inhibits the proliferation of RGCs but promotes the proliferation of IPCs in the developing cortex. Overexpression of *Neurod1* or *Fezf2* in *Arid1a* cKO NSPCs can rescue the neural differentiation defect in vitro. The present study provides direct experimental evidence that *Arid1a* loss of function contributes to abnormal cortical neurogenesis.

In *Arid1a* cKO mice, thinner cortex is detected beginning at E14.5. In consistent with this, the markers of deep‐layer neurons, Tbr1 and Ctip2, are significantly decreased at E16.5 in *Arid1a* cKO mice, wherever the markers of upper‐layer neurons, Brn2 and Satb2, do not significantly changed (Figure 2A‐H). Considering that BAF complex promotes neuronal differentiation in late cortical development,^34^ it is possible that *Arid1a* regulates differentiation of NSPCs along with other BAF subunits. In addition, *Arid1a* deficiency increases cell death in the developing cortex from E12.5 to E16.5. As most TUNEL‐positive cells are located in VZ/SVZ, we speculate that RGCs and/or IPCs are prone to die in *Arid1a* cKO cortex, which should also contribute to the reduced thickness of *Arid1a* cKO cortex. We identify that *Neurod1* and *Fezf2* are functional downstream targets of *Arid1a* to regulate neural differentiation. As currently there is no commercially available ARID1A antibody for chromatin immunoprecipitation analysis of tissues, we analysed the ChIP‐seq and ATAC‐seq data for ARID1A deposited in public databases from embryonic stem cells^30^, ^31^ and retinal ganglion cells^35^ and found that *Arid1a* has enrichment on *Neurod1* and *Fezf2* loci which support our identification ARID1A directly regulates *Neurod1* and *Fezf2* in the nervous system. To our surprise, our results showed that the expression of Brg1, the central ATPase subunit of SWI/SNF, was significantly down‐regulated after *Arid1a* deletion. However, the publicly available ChIP‐seq of BRG1 from E16.5 cortical neurons shows *Brg1* has no enrichment on the same *Neurod1* and *Fezf2* loci, suggesting that Brg1 and Arid1a might have different regulatory mechanisms in neural progenitor/stem cells, while they are the main core components in SWI/SNF complex. The regulatory difference between BRG1 and ARID1A also hints that ARID1A function might be independent of the SWI/SNF complexes in neural progenitor/stem cells or in nervous system. Of course, more investigations are further needed to provide in other systems in the future.

*Arid1a* is a nuclear protein and widely expressed in different human tissues including brain.^36^ Indeed, our study showed that *Arid1a* is ubiquitously expressed in all kinds of brain cells, suggesting its pivotal role not only in NSPCs but also in other cell types. Moreover, higher expression levels of WNT/β‐catenin signal pathway‐associated genes such as *Wnt2b*, *Wnt5a*, *Wnt8b*, *Nfact4* and *Ror2* were observed in *Arid1a* cKO cortex. Given that WNT/β‐catenin signalling is critical for the proper proliferation and differentiation of NSPCs during embryonic development,^37^ combined single‐cell and spatial transcriptomics are required to dissect the complex regulatory network of ARID1A in cortical development.

In summary, this study demonstrates for the first time that *Arid1a* plays an important role in regulating the proliferation and differentiation of NSPCs during cortical development, and proposes several gene candidates that are worth to explore the pathological mechanisms and to develop novel interventions of neurodevelopment diseases caused by *Arid1a* mutations.

## CONFLICT OF INTEREST

The authors declare that they have no conflict of interest.

## AUTHOR CONTRIBUTIONS

C.‐M.L. and X.L. involved in conception and design, collection and assembly of data, data analysis and interpretation, manuscript writing, and final approval of manuscript; S.‐K.D. and P.‐P.L. performed collection and assembly of data.

## Supporting information

Fig S1

Fig S2

Fig S3

Fig S4

Fig S1‐4

Table S1

## Data Availability

The RNA‐seq datasets generated and analysed during the current study have been deposited in the NCBI Sequence Read Archive (SRA). The raw data for E16.5 forebrain RNA‐seq reads are accessible through the series accession numbers PRJNA726035 (https://www.ncbi.nlm.nih.gov/bioproject/PRJNA726035/).
