# Supplementary figures and images for: Arid1a regulates neural stem/progenitor cell proliferation and differentiation during cortical development
Source: Cell Prolif. 2021 Sep 25;54(11):e13124. doi: 10.1111/cpr.13124 (PMC8560606; doi:10.1111/cpr.13124)

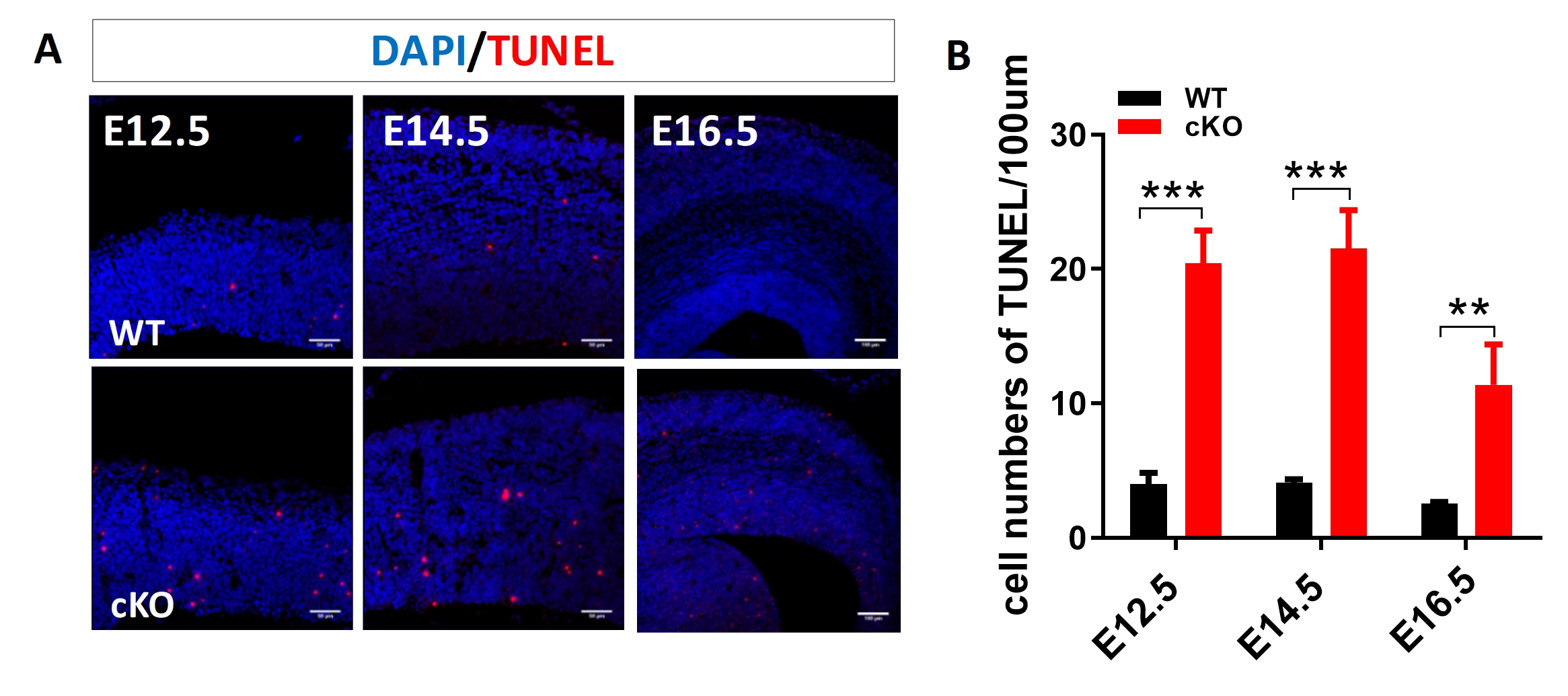

Supplement: Supplementary file 1 — Fig S1 [file CPR-54-e13124-s006.jpg]

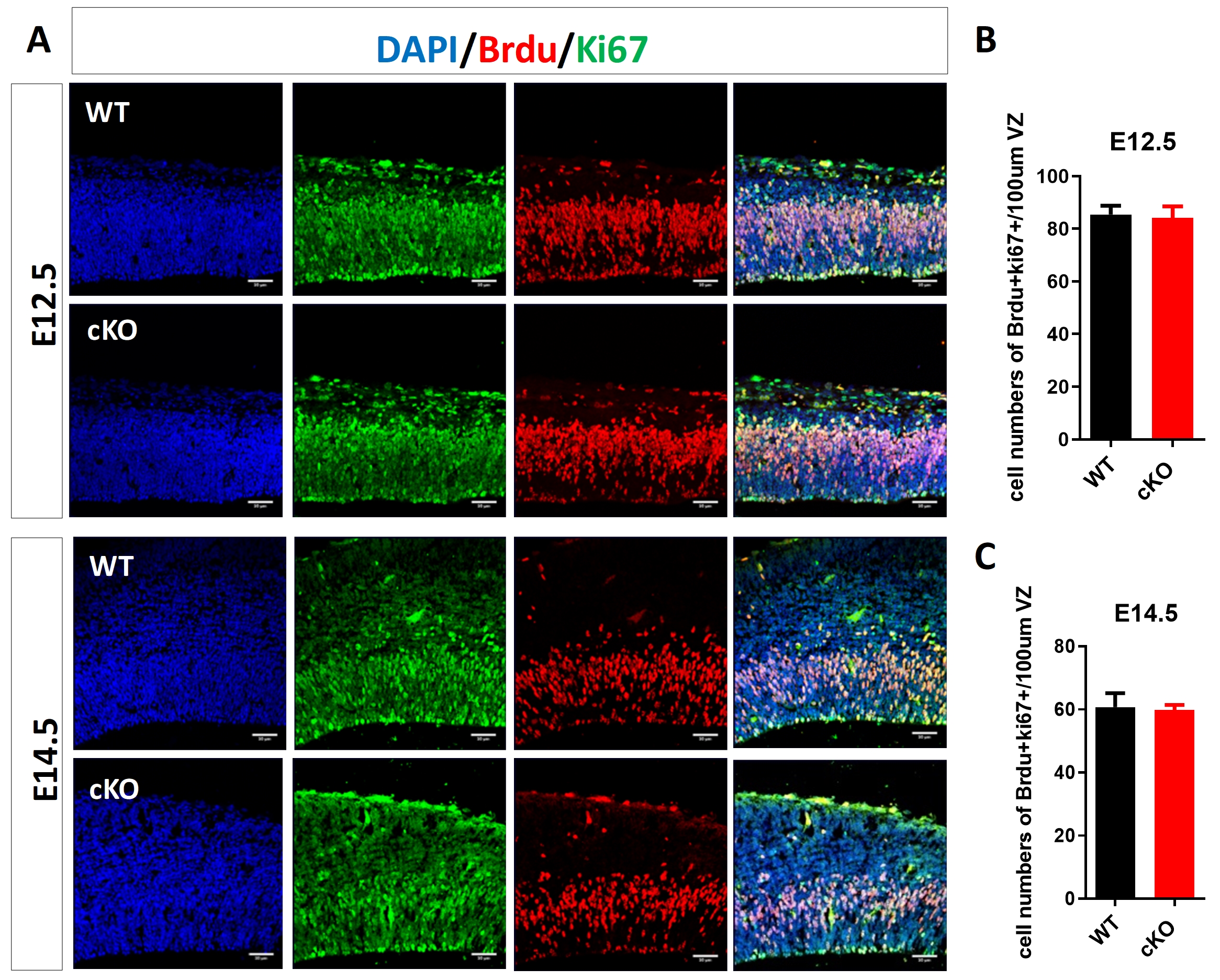

Supplement: Supplementary file 2 — Fig S2 [file CPR-54-e13124-s002.jpg]

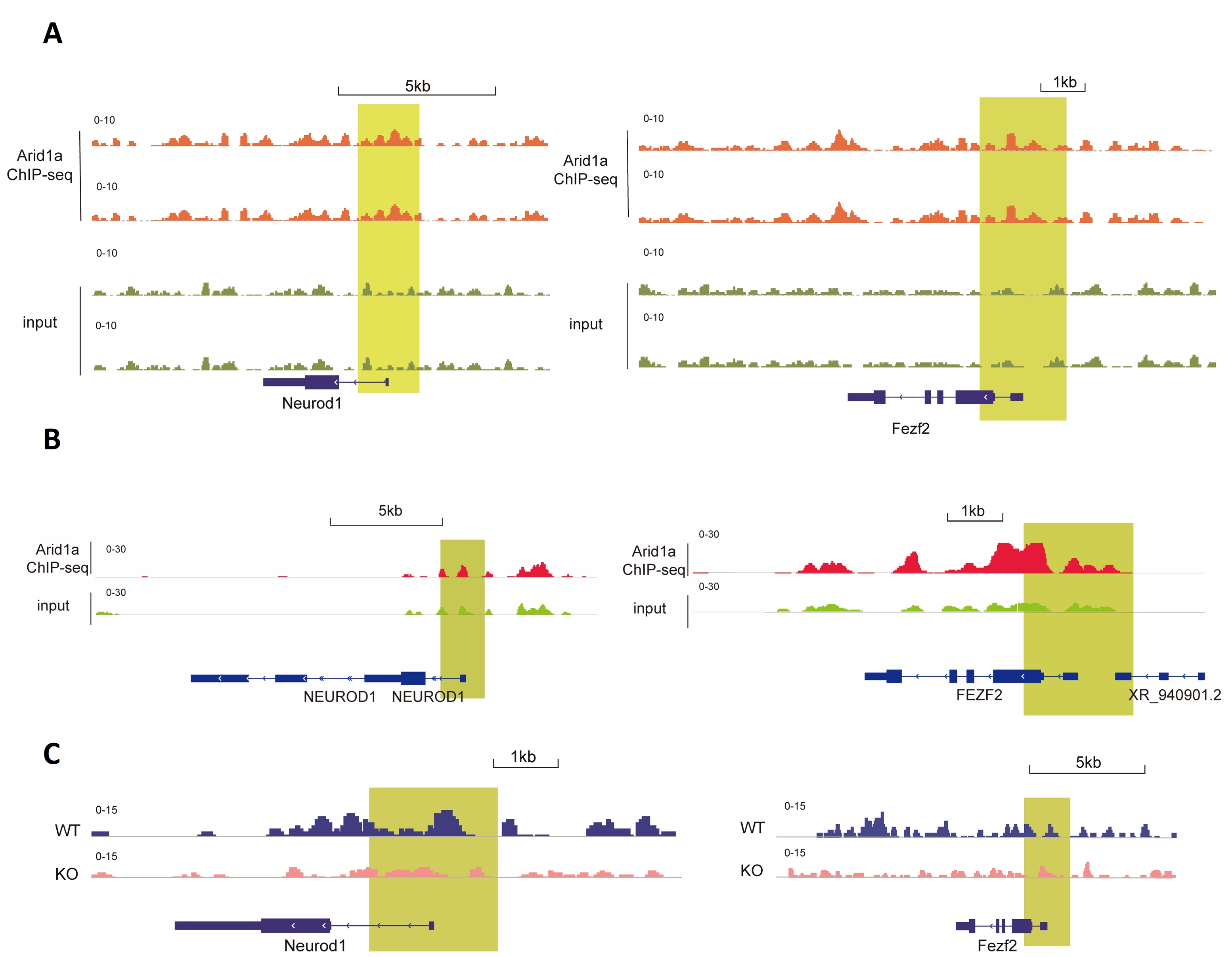

Supplement: Supplementary file 3 — Fig S3 [file CPR-54-e13124-s003.jpg]

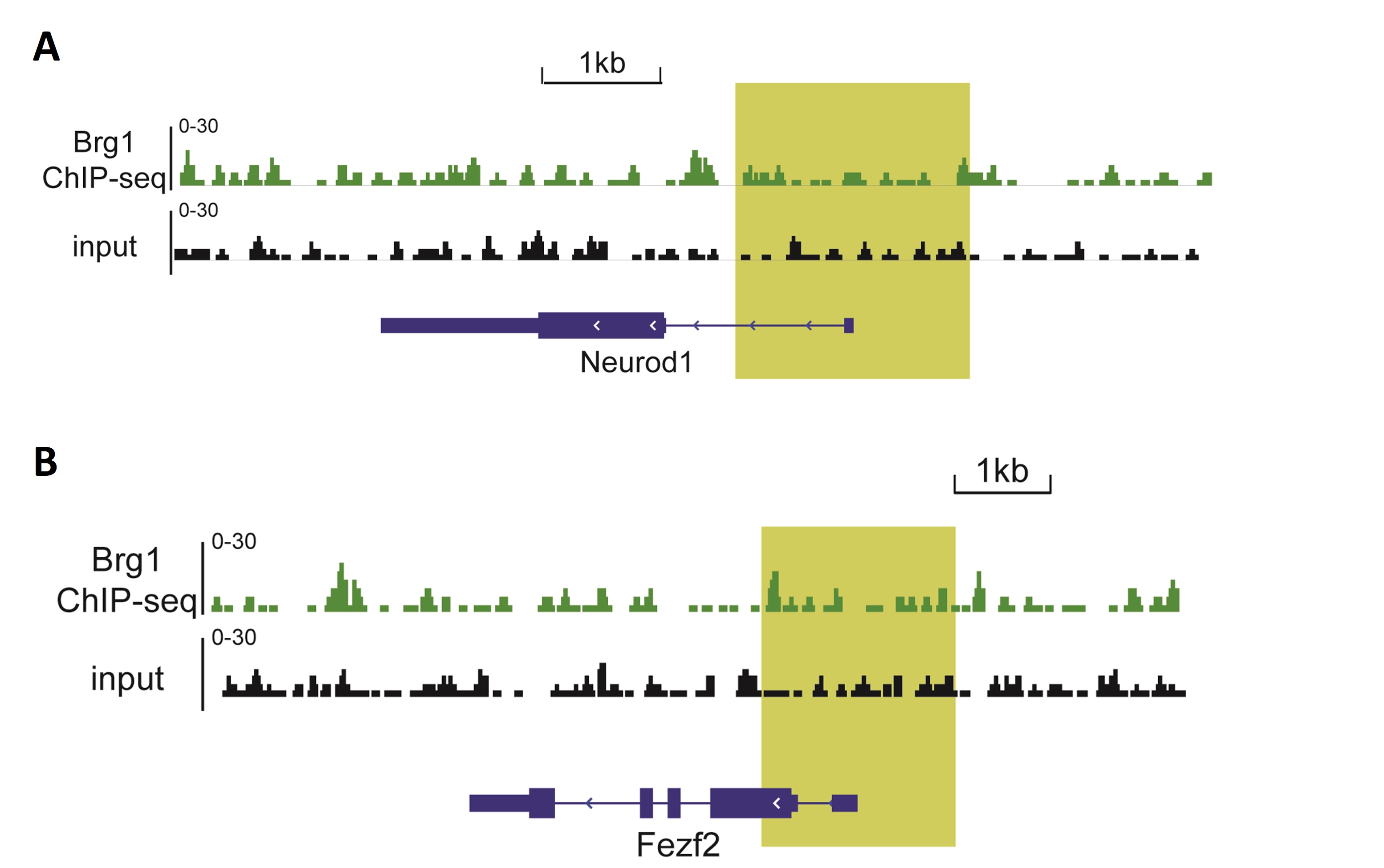

Supplement: Supplementary file 4 — Fig S4 [file CPR-54-e13124-s001.jpg]
