# Supplementary material for: Arid1a regulates neural stem/progenitor cell proliferation and differentiation during cortical development
Source: Cell Prolif. 2021 Sep 25;54(11):e13124. doi: 10.1111/cpr.13124 (PMC8560606; doi:10.1111/cpr.13124)
Supplement: Supplementary file 5 — Fig S1‐4 [file CPR-54-e13124-s004.docx]

**Arid1a regulates neural stem/progenitor cell proliferation and differentiation during cortical development**

Xiao Liu^1, 2, 3,4^, Shang-Kun Dai^1, 2, 3,4^, Pei-Pei Liu^1, 3,4^, Chang-Mei Liu^1, 2, 3,4*^

1. State Key Laboratory of Stem Cell and Reproductive Biology, Institute of Zoology, Chinese Academy of Sciences, Beijing 100101, China.
2. Savaid Medical School, University of Chinese Academy of Sciences, Beijing 100049, China.
3. Institute for Stem Cell and Regeneration, Chinese Academy of Sciences, Beijing 100101, China.
4. Beijing Institute for Stem Cell and Regenerative Medicine, Beijing, China

**Running title:** Arid1a regulates proliferation and differentiation

**Key words:** Cerebral cortex, *Arid1a*, NSPCs, Neurogenesis, Proliferation, Differentiation

* Correspondence author: Chang-Mei Liu ([liuchm@ioz.ac.cn](mailto:liuchm@ioz.ac.cn), 8610-82619690)

**Supplemental Information**


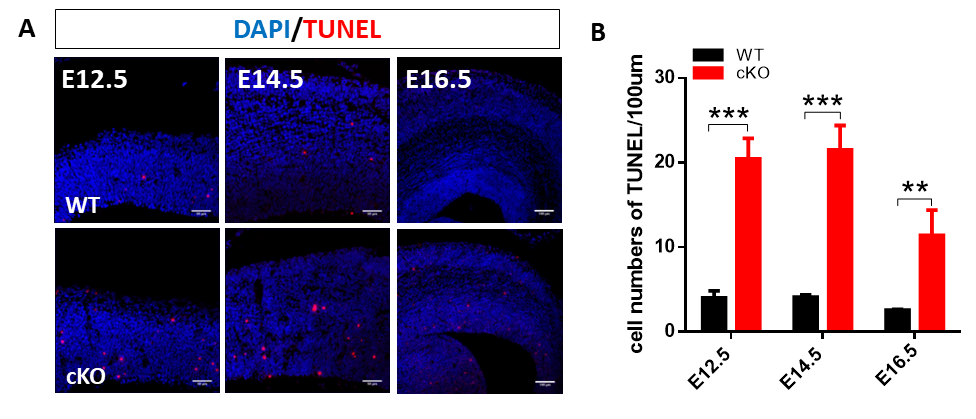


**Figure S1.** Knockout of *Arid1a* leads to increased apoptosis in cerebral cortex. (A-B) TUNEL staining showed that *Arid1a* cKO mice had a significant increase in apoptosis at E12.5, E14.5 and E16.5. WT: n=3, cKO: n=3. Scale bar, 50μm. ***P* < 0.01, ****P* < 0.001.


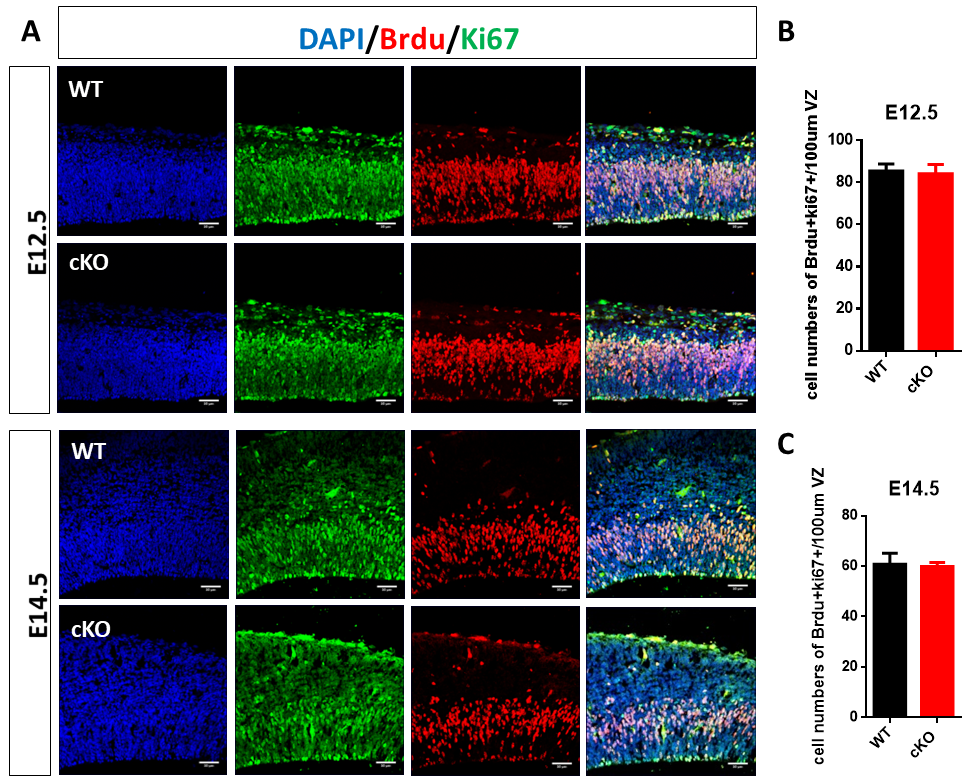


**Figure S2.** *Arid1a* loss-of-function does not impair the proliferation of NSPCs at E12.5 and E14.5. (A) Representative images of BrdU (red) and Ki67 (green) immunofluorescence staining of *Arid1a* WT and cKO brain sections at E12.5 and E14.5. (B-C) Quantitative analysis of BrdU^+^Ki67^+^ cell numbers in the cerebral cortex from *Arid1a* WT and cKO mice at E12.5 and E14.5. WT: n=3; cKO: n=3. Scale bar, 50μm.


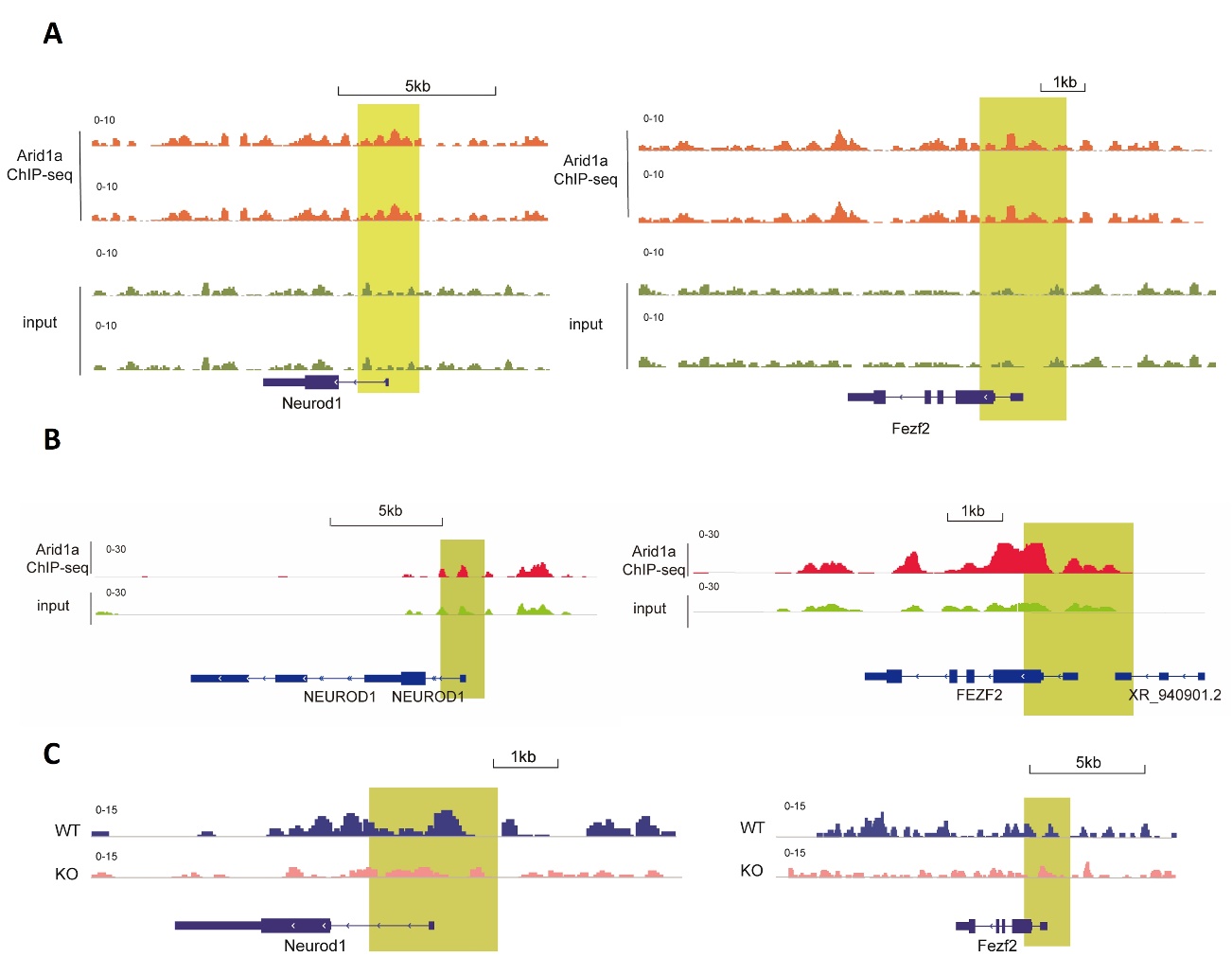


**Figure S3** Arid1a shows stronger enrichment at sites marked with Neurod1 and Fezf2.

(A) Arid1a ChIP-seq analysis of Arid1a occupancy at the Neurod1(left) and Fezf2 (right) promoters (±1000 bp from transcriptional start sites, highlighted in yellow) and intragenic regions was performed in cultured mouse embryonic stem cells. (B) Arid1a ChIP-seq analysis showed stronger enrichment at sites marked with NEUROD1(left) and FEZF2(right) promoters (±1000 bp from transcriptional start sites, highlighted in yellow) and intragenic regions was performed in cultured human embryonic stem cells. (C) Genome views of Neurod1(left) and Fezf2(right) with decreased chromatin accessibility in Arid1a KO retinal ganglion cells compared to WT. Arrows showed the transcriptional direction.


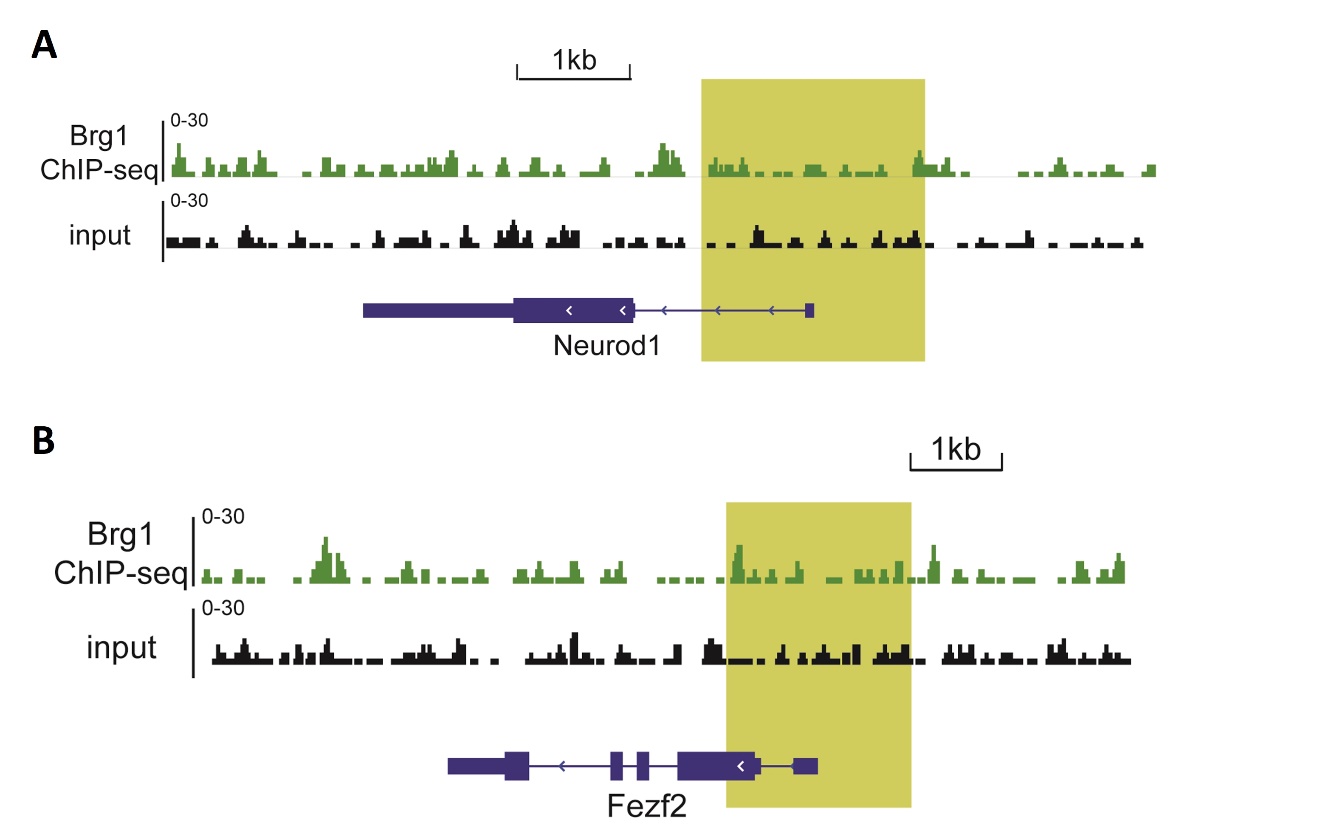


**Figure S4** Brg1 showed no binding enrichment at sites marked with Neurod1 and Fezf2. Genome-wide maps of BRG1 binding sites in cortical neuron under basal and depolarized conditions [ChIP-seq] E16.5 cortical neuron (GSE174581). ChIP-seq analysis of Brg1 occupancy at the Neurod1(A) and Fezf2 (B) promoters (±1000 bp from transcriptional start sites, highlighted in yellow) and intragenic regions was performed in cultured E16.5 cortical neurons from mice.
